# Bayesian and Classical Inference under Type-II Censored Samples of the Extended Inverse Gompertz Distribution with Engineering Applications

**DOI:** 10.3390/e23121578

**Published:** 2021-11-26

**Authors:** Ahmed Elshahhat, Hassan M. Aljohani, Ahmed Z. Afify

**Affiliations:** 1 Faculty of Technology and Development, Zagazig University, Zagazig 44519, Egypt; aelshahhat@ftd.zu.edu.eg; 2 Department of Mathematics & Statistics, College of Science, Taif University, P.O. Box 11099, Taif 21944, Saudi Arabia; hmjohani@tu.edu.sa; 3 Department of Statistics, Mathematics and Insurance, Benha University, Benha 13511, Egypt

**Keywords:** Bayesian estimation, inverse-Gompertz distribution, entropies, moments, stress-strength reliability, maximum likelihood estimation, Type-II censored data, MCMC

## Abstract

In this article, we introduce a new three-parameter distribution called the extended inverse-Gompertz (EIGo) distribution. The implementation of three parameters provides a good reconstruction for some applications. The EIGo distribution can be seen as an extension of the inverted exponential, inverse Gompertz, and generalized inverted exponential distributions. Its failure rate function has an upside-down bathtub shape. Various statistical and reliability properties of the EIGo distribution are discussed. The model parameters are estimated by the maximum-likelihood and Bayesian methods under Type-II censored samples, where the parameters are explained using gamma priors. The performance of the proposed approaches is examined using simulation results. Finally, two real-life engineering data sets are analyzed to illustrate the applicability of the EIGo distribution, showing that it provides better fits than competing inverted models such as inverse-Gompertz, inverse-Weibull, inverse-gamma, generalized inverse-Weibull, exponentiated inverted-Weibull, generalized inverted half-logistic, inverted-Kumaraswamy, inverted Nadarajah–Haghighi, and alpha-power inverse-Weibull distributions.

## 1. Introduction

The two-parameter Gompertz (Go) distribution is very important in modeling actuarial tables and human mortality. It was, historically, introduced by [1], after which many authors have contributed to its statistical methodology and characterization. Several studies have shown that the Go distribution is not flexible for modeling various phenomena due to it having only an increasing hazard rate (HR) shape, for example, the generalized-Go [2], beta-Go [3], transmuted-Go [4], McDonald-Go [5], exponentiated generalized Weibull-Go [6], unit-Go [7], power-Go [8], skew reflected-Go [9], Topp-Leone Go [10], and alpha-power Go [11] distributions.

Furthermore, Wu et al. [12] estimated the parameters of the Go distribution using the least-squares approach. Soliman et al. [13] estimated the parameters of the Go distribution using the maximum likelihood (ML) and Bayes methods under progressive first-failure censored samples. Dey et al. [14] studied the properties and different methods of estimation for the Go distribution.

Recently, many authors have constructed inverted models and studied their applications in several applied fields such as the inverse Nakagami-m, inverse weighted-Lindley, and logarithmic transformed inverse-Weibull distributions by [15,16,17], respectively.

Eliwa et al. [18] proposed the two-parameter inverse Go (IGo) distribution with an upside-down bathtub shape HR function. The non-negative random variable (rv) *X* is said to have an IGo distribution if its cumulative distribution function (CDF) is specified (for x>0) by

(1) $$R\left(x;\beta ,\theta \right)=\exp\left\{-\frac{\beta }{\theta }\left[\exp\left(\frac{\theta }{x}\right)-1\right]\right\},\;\;\beta ,\theta >0,$$

where β and θ denote the shape and scale parameters, respectively.

The first objective of this article is to present a new lifetime model called the EIGo distribution and explore some of its useful properties. Specifically, the EIGo model is constructed based on the extended-R (E-R) family [19] by adding another shape parameter that might address the lack of fit of the IGo distribution for modeling real-life data that indicated non-monotone failure rates. We are motivated to construct the EIGo distribution because (i) it is capable of modelling unimodal HR shape, which provides a good fit for the real data sets; (ii) the EIGo model contains some special well-known distributions; (iii) the EIGo model can be considered as a good alternative to the IGo model and other competing inverted models for fitting the positive data with a longer right tail; and (iv) the EIGo distribution outperforms some competing inverted distributions with respect to two real engineering data sets. One of the important advantages of the EIGo model is its ability to provide an improved fit with respect to its competing inverted models.

The second objective is to address and evaluate the behavior of classical and Bayesian estimators for the unknown parameters of the proposed EIGo distribution under Type-II censored samples. We compare the performances of these estimators by conducting extensive simulations in terms of their root mean squared errors (RMSEs) and relative absolute biases (RABs).

The paper is outlined in seven sections. In Section 2, the EIGo distribution is introduced with its special cases and expansion. Some of its useful properties are addressed in Section 3. In Section 4, maximum likelihood and Bayesian methods are discussed under Type-II censored samples. In Section 5, the performances of the maximum likelihood and Bayesian approaches are explored via simulation results. In Section 6, the adaptability of the EIGo model is addressed using two real-life engineering datasets. Finally, some concluding remarks are presented in Section 7.

## 2. The EIGo Distribution

In this section, we introduce the three-parameter EIGo distribution and some of its sub-models. The CDF of the E-R family, with a shape parameter α>0, has the form

(2) $$F\left(x;\alpha \right)\;=1-{\left[1-R(x)\right]}^{\alpha },\;\;x\in ℜ.$$

A lifetime rv *X* is said to have the EIGo distribution if its CDF has the form

(3) $$F\left(x\right)=1-{\left[1-\exp\left(-\frac{\beta }{\theta }\left(e^{\theta /x\;}-1\right)\right)\right]}^{\alpha },\;x>0,\;\alpha \;\beta ,\;\theta \;>0,$$

where α and β denote the shape parameters and θ denotes the scale parameter. The first advantage of the EIGo distribution is that it has a closed form for its CDF (3).

The corresponding probability density function (PDF) of (3) becomes

(4) $$f\left(x\right)=\alpha \beta x^{-2}\exp\left(\frac{\theta }{x}-\frac{\beta }{\theta }\left(e^{\theta /x\;}-1\right)\right){\left[1-\exp\left(-\frac{\beta }{\theta }\left(e^{\theta /x\;}-1\right)\right)\right]}^{\alpha -1},\;x>0,\;\alpha ,\beta ,\theta >0.$$

The rv with the PDF (4) is denoted by X∼EIGo(α,β,θ). The EIGo distribution involves three well-known lifetime sub-models as follows.

- The generalized inverted-exponential (GIE) distribution [20] follows when the parameter θ→0.
- The IGo distribution [18] is derived for α=1.
- The inverse-exponential (IE) distribution [21] with one parameter β can be derived when θ→0 and α=1.

**Figure 1:**
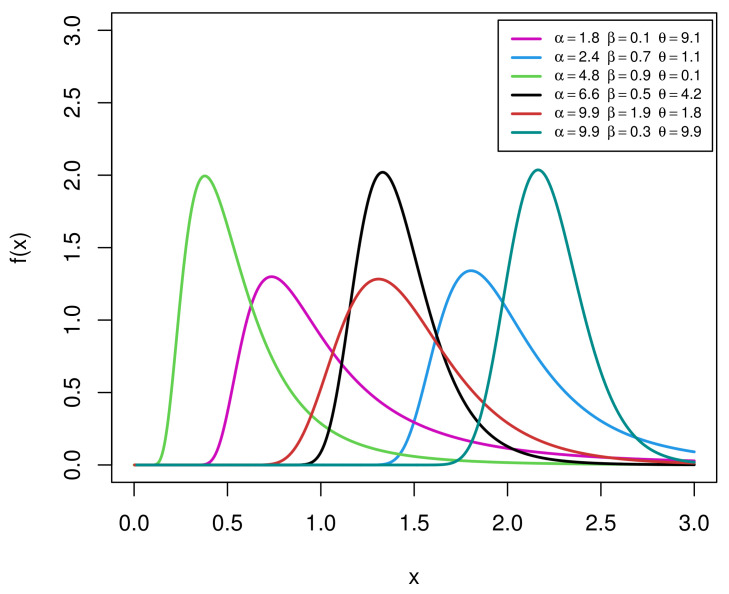
Plots of the PDF of the EIGo distribution for some specific parameter values.

Using some specific parameter values, the shapes of the EIGo PDF (4) are displayed in Figure 1. It shows that the PDF of the EIGo distribution can be unimodal and right-skewed with great heaviness of the tails.

The corresponding survival, S(x), and HR, h(x), functions of the EIGo distribution have the forms

(5) $$S\left(x\right)={\left[1-\exp\left(-\frac{\beta }{\theta }\left(e^{\theta /x\;}-1\right)\right)\right]}^{\alpha },\;x>0$$

and

(6) $$h\left(x\right)=\alpha \beta x^{-2}\exp\left(\frac{\theta }{x}-\frac{\beta }{\theta }\left(e^{\theta /x\;}-1\right)\right){\left[1-\exp\left(-\frac{\beta }{\theta }\left(e^{\theta /x\;}-1\right)\right)\right]}^{-1},\;x>0.$$

**Figure 2:**
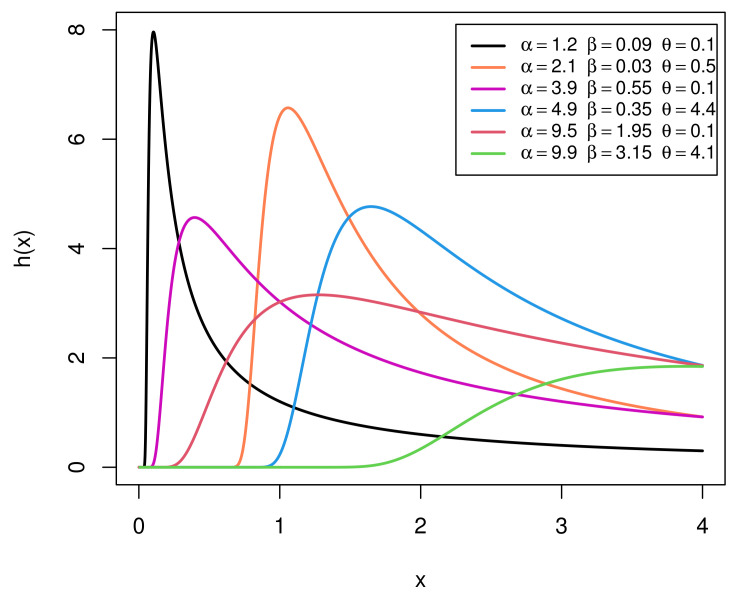
Plots of the HRF of the EIGo distribution for some specific parameter values.

Figure 2 provides graphical representations of the HR function (HRF) of the EIGo distribution with various values of its parameters. It shows that the HRF of the EIGo distribution has an upside-down bathtub shape.

The cumulative HRF, H(x), of the EIGo distribution has the form

$$H\left(x\right)=-\log\left(S(x)\right)=-\alpha \log\left[1-\exp\left(-\frac{\beta }{\theta }\left(e^{\theta /x\;}-1\right)\right)\right],\;x>0.$$

The reversed HRF, r(·) of the EIGo distribution is

$$r\left(x\right)=\frac{\alpha \beta x^{-2}\exp\left(\frac{\theta }{x}-\frac{\beta }{\theta }\left(e^{\theta /x\;}-1\right)\right){\left[1-\exp\left(-\frac{\beta }{\theta }\left(e^{\theta /x\;}-1\right)\right)\right]}^{-1}}{1-{\left[1-\exp\left(-\frac{\beta }{\theta }\left(e^{\theta /x\;}-1\right)\right)\right]}^{\alpha }},\;x>0.$$

By dividing the CDF (3) by the survival function (5), the corresponding odds function, O(x), follows as

$$O\left(x\right)=\frac{1-{\left[1-\exp\left(-\frac{\beta }{\theta }\left(e^{\theta /x\;}-1\right)\right)\right]}^{\alpha }}{{\left[1-\exp\left(-\frac{\beta }{\theta }\left(e^{\theta /x\;}-1\right)\right)\right]}^{\alpha }},\;x>0.$$

### Expansions

Let *a* be a real positive integer; we consider the following general binomial series

(7) $${\left(1-x\right)}^{a}=\sum_{i=0}^{\infty }\frac{{\left(-1\right)}^{i}a!x^{i}}{i!(a-i)!},$$

which is valid for |x|<1. By expanding the CDF (3) by (7), we get

(8) $$F\left(x\right)=1-\sum_{i=0}^{\infty }\vartheta_{i}\left(\alpha \right)\;G^{*}\left(x;i\;\beta ,\theta \right),$$

where $G^{*}\left(x;i\;\beta ,\theta \right)$ is the CDF of the IGo distribution with parameters iβ and θ, and the coefficient $\vartheta_{i}\left(\alpha \right)$ is given by

$$\vartheta_{i}\left(\alpha \right)=\frac{{\left(-1\right)}^{i}}{i!}\frac{\Gamma (\alpha +1)}{\Gamma (\alpha -i+1)!}\cdot$$

Similarly, expanding the PDF (4) by (7), we obtain

(9) $$f\left(x\right)=\alpha \sum_{i=0}^{\infty }\omega_{i}\left(\alpha \right)\;g^{*}\left(x;\left(i+1\right)\;\beta ,\theta \right),$$

where $g^{*}\left(x;\left(i+1\right)\;\beta ,\theta \right)$ is the PDF of the IGo model with parameters (i+1)β and θ, and $\omega_{i}\left(\alpha \right)={\left(-1\right)}^{i}\;\Gamma \left(\alpha \right)/\left[\Gamma \left(\alpha -i\right)\;i!\right]$.

Clearly, Equation (9) shows that the EIGo model is a linear combination of IGo densities. Thus, some structural properties of the EIGo model can be obtained from those of the IGo distribution.

## 3. Statistical and Reliability Characteristics

This section is devoted to determining several statistical and reliability characteristics of the EIGo distribution.

### 3.1. Quantile and Mode

To simulate random samples from the EIGo distribution, its quantile function (QF), $x_{q}$, follows as

(10) $$x_{q}=\frac{\theta }{\ln\left\{1-\frac{\theta }{\beta }\ln\left[1-{\left(1-q\right)}^{1/\alpha \;}\right]\right\}},\;0<q<1.$$

Substituting q=0.5 into (10), the median, Med(x), of the EIGo distribution can conveniently be derived. Similarly, substituting q=0.25 and q=0.75 into (10), the first and the third quartiles of the EIGo distribution can be easily obtained.

The mode of the EIGo distribution follows by differentiating the logarithm of the PDF (4), $x_{0}$, with respect to *x*, and equating the result to zero. After some algebraic manipulations, the mode is determined by solving the following non-linear equation:

(11) $$x_{0}=\beta e^{\theta /x}-2x-\theta -\left(\alpha -1\right)\exp\left(\frac{\theta }{x}-\frac{\beta }{\theta }\left(e^{\theta /x}-1\right)\right){\left[1-\exp\left(-\frac{\beta }{\theta }\left(e^{\theta /x}-1\right)\right)\right]}^{-1}=0.$$

The unique mode of the EIGo distribution cannot be obtained analytically, hence it can be obtained numerically.

### 3.2. Mean Residual Life

The mean-residual-life (MRL) function is the average remaining life span, which is a component surviving up to distinct time *t*. It is a useful measure in reliability studies for describing the aging process.

**Theorem** **1.** *If X has EIGo(α,β,θ) distribution, thus the MRL of the lifetime rv X, say $m_{R}\left(\cdot \right)$, takes the form*

$$m_{R}\left(t\right)=\frac{1}{S\left(t\right)}\left({\mu^{\prime }}_{1}-\sum_{i,s,m=0}^{\infty }\sum_{j=0}^{s}\frac{\alpha !{\left(s-j\right)}^{m}\theta^{m-s}{\left(i\beta \right)}^{s}t^{1-m}}{\left(\alpha -i)!(s-j)!i!j!m!(1-m\right)}{\left(-1\right)}^{i+s+j}\right),\;t>0.$$

**Proof.**  Suppose that *X* is a lifetime rv with CDF (3); then the MRL is defined as [22]

(12) $$m_{R}\left(t\right)=E\left(\left.X-t\right|X>t\right)=\frac{1}{P\left(X>t\right)}\int_{t}^{\infty }\left(x-t\right)dP\left(X⩽x|X>t\right),\;t>0.$$

However, the MRL in (12) is equivalent to

(13) $$\begin{array}{cc} m_{R}\left(t\right) & =\frac{1}{S(t)}\int_{t}^{\infty }S(x)dx,\\ \; & =\frac{1}{S\left(t\right)}\left({\mu^{\prime }}_{1}-\int_{0}^{t}S(x)dx\right),\;t>0, \end{array}$$

where ${\mu^{\prime }}_{1}$ is the expected mean of time *t*, which is equivalent to the MRL at t=0.

Using (5), one gets

(14) $$m_{R}\left(t\right)=\frac{1}{S(t)}\left({\mu^{\prime }}_{1}-\sum_{i,s,m=0}^{\infty }\sum_{j=0}^{s}\vartheta_{i,s,j,m}^{*}\left(\alpha ,\beta ,\theta \right)t^{1-m}\right),$$

where

$$\vartheta_{i,s,j.m}^{*}\left(\alpha ,\beta ,\theta \right)=\frac{\alpha !{\left(s-j\right)}^{m}\theta^{m-s}{\left(i\beta \right)}^{s}}{\left(\alpha -i)!(s-j)!i!j!m!(1-m\right)}{\left(-1\right)}^{i+s+j}.$$

□

### 3.3. Mean Inactivity Time

The mean inactivity time (MIT) function is useful in reliability and survival analysis. The MIT, $m_{I}\left(x\right)$, of *X* is defined as

(15) $$\begin{array}{cc} m_{I}\left(t\right) & =E\left(t-X|X\leq t\right)=\frac{\int_{0}^{t}F\left(x\right)dx}{F\left(t\right)}. \end{array}$$

If X∼EIGo(α,β,θ), then, using (8), we have

(16) $$\int_{0}^{t}F\left(x\right)dx=t-\sum_{i,s,m=0}^{\infty }\sum_{j=0}^{s}\vartheta_{i,s,j,m}^{*}\left(\alpha ,\beta ,\theta \right)t^{1-m}.$$

The MIT of the EIGo distribution follows simply by substituting (3) and (16) in Equation (15). Moreover, the CDF (3) of the EIGo distribution follows from the MIT by the following formula

$$F\left(x\right)=\exp\left(-\int_{x}^{\infty }\frac{1-m_{I}^{\prime }\left(t\right)}{m_{I}\left(t\right)}\right)dt,$$

where $m_{I}\left(t\right)$ is differentiable.

### 3.4. Strong MIT

The strong-MIT (SMIT) function is another useful reliability measure, which is introduced by [23]. They showed that the SMIT has several properties that can be adopted in different applications in reliability and survival analysis. It can be used to predict the actual time at which the failure of the component or device occurs.

The SMIT, $m_{S}\left(x\right)$, has the form

(17) $$m_{S}\left(t\right)\;=\frac{1}{F\left(t\right)}\int_{0}^{t}2xF\left(x\right)dx.$$

Hence, the SMIT of the EIGo distribution follows as

$$m_{S}\left(t\right)\;=\frac{t^{2}}{F(t)}\left(1-2\sum_{i,s,m=0}^{\infty }\sum_{j=0}^{s}\vartheta_{i,s,j,m}^{*}\left(\alpha ,\beta ,\theta \right)\frac{\left(1-m\right)}{\left(2-m\right)}t^{-m}\right).$$

### 3.5. Stress–Strength Reliability

The stress–strength model describes the life of a component or system that has a random strength *X* that may fail because it is subjected to a random stress *Z*. Hence, R=Pr(X>Z) is a measure of component reliability.

Suppose *X* and *Z* have independent EIGo$\left(\alpha ,\beta_{1},\theta_{1}\right)$ and EIGo$\left(\alpha ,\beta_{2},\theta_{2}\right)$ with the same shape parameter α. Then, the stress–strength measure is [24]

(18) $$R=\int_{0}^{\infty }f_{1}\left(x\right)F_{2}\left(x\right)dx.$$

Using (9) and (8), the PDF of $X_{1}$ and the CDF of $X_{2}$ are expressed as

(19) $$f_{1}\left(x\right)=\alpha \sum_{i,s=0}^{\infty }\sum_{j=0}^{s}\frac{\left(\alpha -1\right)!{\left(i+1\right)}^{s}\beta_{1}^{s+1}\theta_{1}^{-s}}{(\alpha -i-1)!(s-j)!i!j!}{\left(-1\right)}^{i+s+j}x^{-2}e^{-\left(j-s-1\right)\theta_{1}/x}$$

and

(20) $$F_{2}\left(x\right)=1-\sum_{u,w=0}^{\infty }\sum_{v=0}^{w}\frac{\alpha !}{(\alpha -u)!(w-v)!u!v!}{\left(-1\right)}^{u+w+v}{\left(\frac{u\beta_{2}}{\theta_{2}}\right)}^{w}e^{\left(w-v\right)\theta_{2}/x\;},$$

respectively.

From substituting (19) and (20) in (18), the *R* measure reduces to

$$R=\alpha \left[\sum_{i,s=0}^{\infty }\sum_{j=0}^{s}\frac{\psi_{i,s,j}^{1}\left(\alpha ,\beta_{1},\theta_{1}\right)}{\left(j-s-1\right)\theta_{1}}-\sum_{i,s,u,w=0}^{\infty }\sum_{j=0}^{s}\sum_{v=0}^{w}\frac{\psi_{i,s,j}^{1}\left(\alpha ,\beta_{1},\theta_{1}\right)\psi_{u,w,v}^{2}\left(\alpha ,\beta_{2},\theta_{2}\right)}{\left(\left(j-s-1\right)\theta_{1}-\left(w-v\right)\theta_{2}\right)}\right],$$

where

$$\psi_{i,s,j}^{1}\left(\alpha ,\beta_{1},\theta_{1}\right)=\frac{\left(\alpha -1\right)!{\left(i+1\right)}^{s}\beta_{1}^{s+1}\theta_{1}^{-s}}{(\alpha -i-1)!(s-j)!i!j!}{\left(-1\right)}^{i+s+j}$$

and

$$\psi_{u,w,v}^{2}\left(\alpha ,\beta_{2},\theta_{2}\right)=\frac{\alpha !u^{w}\beta_{2}^{w}}{\left(\alpha -u\right)!\left(w-v\right)!u!v!\theta_{2}^{w}}{\left(-1\right)}^{u+w+v}.$$

### 3.6. Probability Weighted Moments

The probability-weighted moments (PWM), say $\rho_{r,k}$, can be adopted to derive an estimate for the unknown parameters of a particular distribution whose inverse extension cannot be expressed explicitly.

**Theorem** **2.** *If X has EIGo(α,β,θ) distribution, then the (r,k)th PWM of the rv X is derived as*

(21) $$\rho_{r,k}=\alpha \left[\frac{{\left(-1\right)}^{v+i+s+j}\Gamma \left(k+1\right)\Gamma \left(\alpha \left(v+1\right)\right)\Gamma \left(m-r+1\right){\left(i+1\right)}^{s}\beta^{s+1}\theta^{r-s-1}{\left(j-s\right)}^{r-m-1}}{\Gamma (k-v+1)\Gamma (\alpha (v+1)-i)\Gamma (s-j+1)\Gamma (v+1)\Gamma (i+1)\Gamma (s+1)\Gamma (j+1)}\right]\cdot$$

**Proof.**  The (r,k)th PWM of non-negative *X* following a continuous CDF, F(.), is defined by [25]

(22) $$\rho_{r,k}=E\left[X^{r}F^{k}\left(x\right)\right]=\int_{0}^{\infty }x^{k}F^{k}\left(x\right)f\left(x\right)dx.$$

Substituting (3) and (4) into (22), the $\rho_{r,k}$ can be written as

(23) $$\begin{array}{cc} \rho_{r,k}=\int_{0}^{\infty }x^{k}F^{k}\left(x\right)\;f\left(x\right)\;dx & =\alpha \;\beta \;\sum_{v,i=0}^{\infty }\frac{{\left(-1\right)}^{v+i}\;\Gamma \left(k+1\right)\;\Gamma \left(\alpha \;\left(v+1\right)\right)}{\Gamma (i+1)\;\Gamma (k-v+1)\;\Gamma (\alpha (v+1)-i)\;\Gamma (v+1)}\\ \; & \times \int_{0}^{\infty }x^{k-2}e^{\theta /x}\exp\left(-\frac{(i+1)\beta }{\theta }\left(e^{\theta /x}-1\right)\right)dx, \end{array}$$

The following term can be expanded by Taylor’s series as

$$\exp\left(-\frac{(i+1)\;\beta }{\theta }\left(e^{\theta /x}-1\right)\right)=\sum_{s=0}^{\infty }{\left(-1\right)}^{s}\frac{{\left(i+1\right)}^{s}\beta^{s}}{\theta^{s}\;\Gamma \left(s+1\right)}{\left(e^{\theta /x}-1\right)}^{s}.$$

Hence, Equation (23) reduces to

(24) $$\begin{array}{cc} \rho_{r,k} & =\alpha \sum_{v,i,s=0}^{\infty }\frac{{\left(-1\right)}^{v+i+s}\Gamma \left(k+1\right)\;\Gamma \left(\alpha \;\left(v+1\right)\right)\;{\left(i+1\right)}^{s}\beta^{s+1}}{\Gamma \left(k-v+1\right)\;\Gamma \left(\alpha \left(v+1\right)-i\right)\;\Gamma \left(v+1\right)\;\Gamma \left(i+1\right)\;\Gamma \left(s+1\right)\theta^{s}}\\ \; & \times \int_{0}^{\infty }x^{k-2}e^{\theta /x}{\left(e^{\theta /x}-1\right)}^{s}dx. \end{array}$$

However, from (24), the $\rho_{r,k}$ of *X* has the form

$$\rho_{r,k}=\alpha \;\sum_{v,i,s,m=0}^{\infty }\sum_{j=0}^{s}\upsilon_{v,i,s,m,j}\left(\alpha ,\beta ,\theta \right)\;\Gamma \left(m-r+1\right),$$

where

$$\upsilon_{v,i,s,m,j}\left(\alpha ,\beta ,\theta \right)=\frac{{\left(-1\right)}^{v+i+s+j}\;\Gamma \left(k+1\right)\;\Gamma \left(\alpha \left(v+1\right)\right)\;{\left(i+1\right)}^{s}\;\beta^{s+1}\theta^{r-s-1}{\left(j-s\right)}^{r-m-1}}{\Gamma (j+1)\;\Gamma (k-v+1)\;\Gamma (\alpha (v+1)-i)\;\Gamma (s-j+1)\;\Gamma (v+1)\;\Gamma (i+1)\;\Gamma (s+1)}\cdot$$

□

### 3.7. Moments

Moments are used to describe the characteristics of the probability distribution, so they are important in any statistical analysis.

By definition, the *r*-th moment of any rv *X* with PDF, f(x), is

(25) $$\mu_{r}^{{}^{\prime }}=E\left(X^{r}\right)=\int_{0}^{\infty }x^{r}f\left(x\right)\;dx.$$

By substituting (9) in (25), the *r*th moment of the EIGo(α,β,θ) distribution reduces to

(26) $$\begin{array}{cc} \mu_{r}^{{}^{\prime }} & =\alpha \sum_{i,s,m=0}^{\infty }\sum_{j=0}^{s}\frac{\left(\alpha -1\right)!{\left(i+1\right)}^{s}\beta^{s+1}\theta^{m-s}}{(\alpha -i-1)!(s-j)!i!j!m!}{\left(-1\right)}^{i+s+j}\int_{0}^{\infty }x^{r-m-2}e^{-(j-s)\theta /x}dx,\\ \; & =\alpha \sum_{i,s,m=0}^{\infty }\sum_{j=0}^{s}\varpi_{i,s,m,j}\left(\alpha ,\beta ,\theta \right)\Gamma \left(m-r+1\right), \end{array}$$

where

$$\varpi_{i,s,m,j}\left(\alpha ,\beta ,\theta \right)=\frac{\left(\alpha -1\right)!{\left(i+1\right)}^{s}{\left(j-s\right)}^{r-m-1}\beta^{s+1}\theta^{r-s-1}}{(\alpha -i-1)!(s-j)!i!j!m!}{\left(-1\right)}^{i+s+j},$$

$\Gamma \left(a\right)=\int_{0}^{\infty }t^{a-1}e^{-t}dt,\;a>0$ is the gamma (Ga) function.

From (26), the corresponding mean of the EIGo distribution is simply obtained by setting r=1, and the corresponding variance can be also obtained using r=1 and r=2.

### 3.8. Entropies

Entropy is a useful concept to measure the uncertainty related to a rv *X*. It is adopted in many fields of science such as econometrics and computer science.

The Rényi entropy of order δ, say $\rho_{\delta }\left(X\right)$, is defined (for δ>0 and δ≠1) as [26]

$$\rho_{\delta }\left(X\right)=\frac{1}{1-\delta }\log\left(\int_{-\infty }^{\infty }{f\left(x\right)}^{\delta }\;dx\right).$$

So, if X∼EIGo(α,β,θ) distribution, then we have

(27) $$\begin{array}{cc} \int_{0}^{\infty }{\left(f(x)\right)}^{\delta }dx & =\alpha^{\delta }\sum_{i,s=0}^{\infty }\sum_{j=0}^{s}\frac{\left((\alpha -1)\;\delta \right)!\;{\left(i+\delta \right)}^{s}\beta^{s+\delta }\theta^{-s}}{(\delta (\alpha -1)-i-1)!i!s!j!}{\left(-1\right)}^{i+s+j}\int_{0}^{\infty }x^{-2\delta }e^{-\left(j-s-\delta \right)\theta /x\;}dx,\\ \; & =\alpha^{\delta }\sum_{i,s=0}^{\infty }\sum_{j=0}^{s}\omega_{i,s,j}^{\rho_{\delta }}\left(\alpha ,\beta ,\theta \right)\Gamma \left(2\delta -1\right), \end{array}$$

where

$$\begin{array}{c} \omega_{i,s,j}^{\rho_{\delta }}\left(\alpha ,\beta ,\theta \right)=\frac{\left(\delta (\alpha -1)\right)!{\left(i+\delta \right)}^{s}{\left(j-s-\delta \right)}^{1-2\delta }\beta^{s+\delta }\theta^{1-s-2\delta }}{(\delta (\alpha -1)-i-1)!i!s!j!}{\left(-1\right)}^{i+s+j}. \end{array}$$

Hence, the $\rho_{\delta }\left(X\right)$ of *X* becomes

$$\begin{array}{c} \rho_{\delta }\left(X\right)=\frac{1}{1-\delta }\log\left(\alpha^{\delta }\sum_{i,s=0}^{\infty }\sum_{j=0}^{s}\omega_{i,s,j}^{\rho_{\delta }}\left(\alpha ,\beta ,\theta \right)\Gamma \left(2\delta -1\right)\right),\;\delta >0,\;\delta \neq 1. \end{array}$$

The δ-entropy, denoted by $I_{\delta }\left(X\right)$, of the EIGo distribution has the form (for δ>0 and δ≠1)

$$\begin{array}{c} I_{\delta }\left(X\right)=\frac{1}{\delta -1}\log\left(1-\int_{0}^{\infty }{\left(f\left(x\right)\right)}^{\delta }dx\right). \end{array}$$

It follows directly using (27).

### 3.9. Order Statistics

Consider the order statistics (OS) of a random sample of size *n*, say $X_{\left(1\right)}⩽X_{\left(2\right)}⩽\ldots ⩽X_{\left(n\right)}$. Then, the PDF of the r−th order statistic, say $X_{\left(r\right)},\;r=1,2,\ldots ,n$ is [27]

(28) $$f_{\left(r\right)}\left(x\right)=C_{r}^{-1}\sum_{q=0}^{n-r}{\left(-1\right)}^{q}\left(\begin{array}{c} n-r\\ q \end{array}\right)f\left(x\right){\left(F\left(x\right)\right)}^{r+q-1},$$

where $C_{r}=B\left(r,n-r+1\right)\;$.

The corresponding CDF of $X_{\left(r\right)}$ reduces to

(29) $$F_{\left(r\right)}\left(x\right)=\sum_{d=r}^{n}\sum_{q=0}^{n-d}{\left(-1\right)}^{q}\left(\begin{array}{c} n\\ d \end{array}\right)\left(\begin{array}{c} n-d\\ q \end{array}\right){\left(F\left(x\right)\right)}^{d+q},$$

Using (3) and (4) of the EIGo distribution, the PDF (28) follows as

(30) $$f_{\left(r\right)}\left(x\right)=\sum_{q=0}^{n-r}\sum_{v,s=0}^{\infty }\;\xi_{q,v,s}^{\left(r\right)}\left(\alpha ,\beta ,\theta \right)g_{r}^{*}\left(\left(s+1\right)\beta ,\theta ;x\right),$$

where

$$\xi_{q,v,s}^{\left(r\right)}\left(\alpha ,\beta ,\theta \right)=\frac{\alpha \left(n-r\right)!\left(r+q-1\right)!\left(\alpha \left(v+1\right)-1\right)!{\left(-1\right)}^{q+v+s}}{C_{r}\left(m+1\right)\left(n-r-q\right)!\left(r+q-v-1\right)!\left(\alpha \left(v+1\right)-m-1\right)!q!v!s!},$$

and $g_{r}^{*}\left(\left(s+1\right)\beta ,\theta ;x\right)$ is the PDF of the IGo model with parameters (s+1)β and θ. Thus, the PDF of the EIGo distribution OS is a linear mixture of the IGo densities.

Similarly, from using (3) and (4) of the EIGo distribution, the CDF (29) reduces to

(31) $$F_{\left(r\right)}\left(x\right)=\sum_{d=r}^{n}\sum_{q=0}^{n-d}\sum_{q=0}^{n-r}\sum_{v,s=0}^{\infty }\;\zeta_{q,v,s}^{\left(r\right)}\left(\alpha ,\beta ,\theta \right)G_{r}^{*}\left(s\beta ,\theta ;x\right),$$

where

$$\zeta_{d,q,v,s}^{\left(r\right)}\left(\alpha ,\beta ,\theta \right)=\frac{n!\;\left(n-d\right)!\;\left(d+q\right)!\;\left(\alpha v\right)!\;{\left(-1\right)}^{q+v+s}}{(n-d-q)!\;(n-d)!\;(d+q-v)!\;(\alpha v-s)\;!d!\;q!\;v!\;s!}.$$

### 3.10. Stochastic Ordering

The following theorem shows that the EIGo distribution is ordered with respect to the likelihood ratio, $X\leq_{LR}Y$, order.

**Theorem** **3.** *Let X∼EIGo$\left(\alpha_{1},\beta ,\theta \right)$ and Y∼EIGo$\left(\alpha_{2},\beta ,\theta \right)$, for $\alpha_{1}\geq \alpha_{2}$, then $X\leq_{lr}Y$.*

**Proof.**  The likelihood ratio function $\xi \left(x\right)$ has the form (see [28])

(32) $$\xi \left(x\right)=\frac{f_{X}\left(x\right)}{f_{Y}\left(x\right)}.$$

By substituting (3) into (32), one gets

(33) $$\xi \left(x\right)=\frac{\alpha_{1}{\left[1-\exp\left(-\frac{\beta }{\theta }\left(e^{\theta /x\;}-1\right)\right)\right]}^{\alpha_{1}}}{\alpha_{2}{\left[1-\exp\left(-\frac{\beta }{\theta }\left(e^{\theta /x\;}-1\right)\right)\right]}^{\alpha_{2}}}\cdot$$

Taking the natural logarithm of (33) and differentiating the result with respect to *x*, we obtain

$$\frac{d}{dx}\log\left(\xi \left(x\right)\right)=\frac{\left(\alpha_{2}-\alpha_{1}\right)\beta }{x^{2}}\exp\left(\frac{\theta }{x}-\frac{\beta }{\theta }\left(e^{\theta /x\;}-1\right)\right)<0.$$

Thus, $\xi \left(x\right)$ is decreasing in *x* for $\alpha_{1}\geq \alpha_{2}$; i.e., $X\leq_{lr}Y$. The proof is completed. □

## 4. Parameter Estimation under Type-II Censoring

In this section, we discuss the estimation of the EIGo parameters, $\Theta ={\left(\alpha ,\beta ,\theta \right)}^{T}$, using the ML and Bayesian estimators under Type-II censoring scheme in which the life-test is terminated after a specified number of failures have occurred, say m(<n), out of complete test units *n*.

### 4.1. Maximum Likelihood Estimators

Suppose that *n* independent items are taken from the EIGo model with CDF (3) and are placed on a test at time 0. Hence, the likelihood function (LF), say L(Θ|x), under Type-II censored sample, $X_{\left(i\right)},\;i=1,2,...,m$, takes the form (See [29])

(34) $$L\left(\Theta |x\right)=\frac{n!}{(n-m)!}\underset{i=1}{\overset{m}{\Pi }}\;f\left(x_{\left(i\right)};\Theta \right){\left[1-F(x_{\left(m\right)};\Theta )\right]}^{n-m}.$$

By substituting (3) and (4) into (34), then Equation (34) reduces to

(35) $$\begin{array}{cc} L\left(\Theta |x\right) & =\frac{n!{\left(\alpha \beta \right)}^{m}}{(n-m)!}\exp\left(\theta \sum_{i=1}^{m}x_{\left(i\right)}^{-1}\right)\underset{i=1}{\overset{m}{\Pi }}\;x_{\left(i\right)}^{-2}{\left[1-\exp\left(-\zeta \left(x_{\left(i\right)};\beta ,\theta \right)\right)\right]}^{-1}\\ \; & \times \exp\left(\alpha \left(\left(n-m\right)\log\left[1-\exp\left(-\zeta \left(x_{\left(m\right)};\beta ,\theta \right)\right)\right]+\sum_{i=1}^{m}\log\left[1-\exp\left(-\zeta \left(x_{\left(i\right)};\beta ,\theta \right)\right)\right]\right)\right), \end{array}$$

where $\zeta \left(x_{\left(i\right)};\beta ,\theta \right)=\frac{\beta }{\theta }\left(\exp(\theta x_{\left(i\right)}^{-1})-1\right)$ and $\zeta \left(x_{\left(m\right)};\beta ,\theta \right)=\frac{\beta }{\theta }\left(\exp(\theta x_{\left(m\right)}^{-1})-1\right)$. Clearly, the LF of complete sample follows as a special case from (35) by setting m=n. The associated log-LF of (35), say ℓ(Θ), becomes

(36) $$\begin{array}{cc} \ell (\left.\alpha ,\beta ,\theta \right|x) & \propto m\log\left(\alpha \beta \right)+\theta \sum_{i=1}^{m}\left(x_{\left(i\right)}^{-1}\right)+\left(\alpha -1\right)\sum_{i=1}^{m}\log\left[1-\exp\left(-\zeta \left(x_{\left(i\right)};\beta ,\theta \right)\right)\right]\\ \; & +\alpha \left(n-m\right)\log\left[1-\exp\left(-\zeta \left(x_{\left(m\right)};\beta ,\theta \right)\right)\right]. \end{array}$$

Differentiating (36) partially with respect to α, β and θ, we can write that the log-LF of (35), say ℓ(Θ), becomes

(37) $$\begin{array}{c} \frac{\partial \ell (\Theta )}{\partial \alpha }=\frac{m}{\alpha }+\left(n-m\right)\log\left[1-\exp\left(-\zeta \left(x_{\left(m\right)};\beta ,\theta \right)\right)\right]+\sum_{i=1}^{m}\log\left[1-\exp\left(-\zeta \left(x_{\left(i\right)};\beta ,\theta \right)\right)\right], \end{array}$$

(38) $$\begin{array}{cc} \frac{\partial \ell (\Theta )}{\partial \beta } & =\frac{m}{\beta }+\frac{\alpha \left(n-m\right)\zeta_{\beta }^{{}^{\prime }}\left(x_{\left(m\right)};\beta ,\theta \right)\exp\left(-\zeta \left(x_{\left(m\right)};\beta ,\theta \right)\right)}{1-\exp\left(-\zeta \left(x_{\left(m\right)};\beta ,\theta \right)\right)}\\ \; & +\left(\alpha -1\right)\sum_{i=1}^{m}\frac{\zeta_{\beta }^{{}^{\prime }}\left(x_{\left(i\right)};\beta ,\theta \right)\exp\left(-\zeta \left(x_{\left(i\right)};\beta ,\theta \right)\right)}{1-\exp\left(-\zeta \left(x_{\left(i\right)};\beta ,\theta \right)\right)} \end{array}$$

and

(39) $$\begin{array}{cc} \frac{\partial \ell (\Theta )}{\partial \theta } & =\sum_{i=1}^{m}\left(x_{\left(i\right)}^{-1}\right)+\frac{\alpha \left(n-m\right)\zeta_{\theta }^{{}^{\prime }}\left(x_{\left(m\right)};\beta ,\theta \right)\exp\left(-\zeta \left(x_{\left(m\right)};\beta ,\theta \right)\right)}{1-\exp\left(-\zeta \left(x_{\left(m\right)};\beta ,\theta \right)\right)}\\ \; & +\left(\alpha -1\right)\sum_{i=1}^{m}\frac{\zeta_{\theta }^{{}^{\prime }}\left(x_{\left(i\right)};\beta ,\theta \right)\exp\left(-\zeta \left(x_{\left(i\right)};\beta ,\theta \right)\right)}{1-\exp\left(-\zeta \left(x_{\left(i\right)};\beta ,\theta \right)\right)}, \end{array}$$

where $\zeta_{\phi }^{{}^{\prime }}\left(\cdot \right)$ is the first-partial derivative with respect to ϕ, $\zeta_{\beta }^{{}^{\prime }}\left(x_{\left(i\right)};\beta ,\theta \right)=\frac{\zeta (x_{\left(i\right)};\beta ,\theta )}{\beta }$, $\zeta_{\beta }^{{}^{\prime }}\left(x_{\left(m\right)};\beta ,\theta \right)=\frac{\zeta (x_{\left(m\right)};\beta ,\theta )}{\beta }$, $\zeta_{\theta }^{{}^{\prime }}\left(x_{\left(i\right)};\beta ,\theta \right)=\frac{\beta }{\theta^{2}}\left[e^{\theta x_{\left(i\right)}^{-1}}\left(\theta x_{\left(i\right)}^{-1}-1\right)+1\right]$, and $\zeta_{\theta }^{{}^{\prime }}\left(x_{\left(m\right)};\beta ,\theta \right)=\frac{\beta }{\theta^{2}}\left[e^{\theta x_{\left(m\right)}^{-1}}\left(\theta x_{\left(m\right)}^{-1}-1\right)+1\right]$.

Equating the three Equations (37)–(39) to zero and solving them simultaneously will provide the ML estimators (MLEs) of the EIGo parameters. Clearly, the MLEs cannot be determined in closed forms, but they can be calculated numerically using suitable iterative techniques such as the Newton–Raphson. To construct the confidence intervals (CIs) of the model parameters, the observed information matrix, $I_{ij}\left(\cdot \right)$, is required, and it takes the form

(40) $$I_{ij}\left(\underline{\Theta }\right)=E\left[-\left(\partial^{2}\ell \left(\left.\underline{\Theta }\right|x\right)/\partial {\underline{\Theta }}^{2}\;\right)\right],\;i,j=1,2,3.$$

Practically, by dropping the expectation operator given in (40) and replacing $\underline{\Theta }$ by their MLEs $\underline{\hat{\Theta }}$, the approximate asymptotic variance–covariance matrix, $I^{-1}\left(\underline{\hat{\Theta }}\right)$, for the MLEs $\underline{\hat{\Theta }}={\left(\hat{\alpha },\hat{\beta },\hat{\theta }\right)}^{T}$, becomes

(41) $$I^{-1}\left(\underline{\hat{\Theta }}\right)={\left[\begin{array}{cc} \; & -L_{\alpha \alpha }-L_{\alpha \beta }-L_{\alpha \theta }\\ \; & -L_{\beta \alpha }-L_{\beta \beta }-L_{\beta \theta }\\ \; & -L_{\theta \alpha }-L_{\theta \beta }-L_{\theta \theta } \end{array}\right]}_{\left(\underline{\Theta }=\underline{\hat{\Theta }}\right)}^{-1}=\left[\begin{array}{cc} \; & {\hat{\sigma }}_{\hat{\alpha }\hat{\alpha }}\;{\hat{\sigma }}_{\hat{\alpha }\hat{\beta }}\;{\hat{\sigma }}_{\hat{\alpha }\hat{\theta }}\\ \; & {\hat{\sigma }}_{\hat{\beta }\hat{\alpha }}\;{\hat{\sigma }}_{\hat{\beta }\hat{\beta }}\;{\hat{\sigma }}_{\hat{\beta }\hat{\theta }}\\ \; & {\hat{\sigma }}_{\hat{\theta }\hat{\alpha }}\;{\hat{\sigma }}_{\hat{\theta }\hat{\beta }}\;{\hat{\sigma }}_{\hat{\theta }\hat{\theta }} \end{array}\right].$$

Taking the second partial derivative of (36) with respect to α, β, and θ, the observed Fisher elements in (41), $L_{ij}$, are obtained and are available with the authors upon request. Under some mild regularity conditions, the MLEs $\underline{\hat{\Theta }}$ are approximately distributed as multivariate normal (No) distribution with mean $\underline{\Theta }$ and variance $I^{-1}\left(\underline{\hat{\Theta }}\right)$ respectively [29]. Hence, for large samples, 100(1−ε)% CIs for the model parameters α,β and θ are

$$\hat{\alpha }\mp z_{\varepsilon /2\;}\sqrt{{\hat{\sigma }}_{\hat{\alpha }\hat{\alpha }}},\;\hat{\beta }\mp z_{\varepsilon /2\;}\sqrt{{\hat{\sigma }}_{\hat{\beta }\hat{\beta }}}\;\mathrm{and}\;\hat{\theta }\mp z_{\varepsilon /2\;}\sqrt{{\hat{\sigma }}_{\hat{\theta }\hat{\theta }}},$$

respectively, where ${\hat{\sigma }}_{\hat{\alpha }\hat{\alpha }}$, ${\hat{\sigma }}_{\hat{\beta }\hat{\beta }}$, and ${\hat{\sigma }}_{\hat{\theta }\hat{\theta }}$ refer to diagonal elements of (41) and $z_{\varepsilon /2\;}$ is the percentile of the standard No distribution with right-tail probability (ε/2)-th.

### 4.2. Bayes Estimators

This subsection discusses the Bayes estimators (BEs) and the credible intervals (CIs) of the unknown parameters of the EIGo model. The squared error loss (SEL) function as a symmetric loss function is adopted to obtain the BEs, and it is defined by

(42) $$l\left(\Theta ,\tilde{\Theta }\right)={\left(\tilde{\Theta }-\Theta \right)}^{2},$$

where $\tilde{\Theta }$ is an estimate of Θ.

The gamma (Ga) conjugate priors of the EIGo parameters can be applied to develop the BEs due to their flexibility in covering several varieties of prior beliefs of the experimenter (see [30,31]). Hence, the unknown parameters α, β and θ are assumed to have independent Ga prior PDF, i.e., as α∼Ga$\left(a_{1},b_{1}\right)$, β∼Ga$\left(a_{2},b_{2}\right)$ and θ∼Ga$\left(a_{3},b_{3}\right)$. The hyper-parameters, say $a_{i},b_{i}>0,\;i=1,2,3$, represent the prior knowledge about the three parameters, and they are assumed to be non-negative and known. However, the hyper-parameters are fixed by using the mean and the variance of the Ga distribution ($a_{i}=\frac{{\Theta^{2}}_{i}}{\tau^{2}}$ and $b_{2}={\Theta^{2}}_{i}\left(a_{i}-1\right)$); hence, $\tau^{2}=1$ is used and $\Theta_{i}$ is the initial value. Hence, the joint prior PDF of Θ becomes

(43) $$\pi \left(\alpha ,\beta ,\theta \right)\propto \alpha^{a_{1}-1}\beta^{a_{2}-1}\theta^{a_{3}-1}\exp\left(-\left(b_{1}\alpha +b_{2}\beta +b_{3}\theta \right)\right),\;\alpha ,\beta ,\theta >0.$$

Combining (43) with (35), the joint posterior distribution of α, β and θ becomes

(44) $$\begin{array}{cc} \pi (\alpha ,\beta ,\theta |x) & =A^{-1}\alpha^{m+a_{1}-1}e^{-\alpha b_{1}^{*}\left(\beta ,\theta \right)}\beta^{m+a_{2}-1}e^{-b_{2}\beta }\theta^{a_{3}-1}\exp\left(-\theta \left(b_{3}-\sum_{i=1}^{m}x_{\left(i\right)}^{-1}\right)\right)\\ \; & \times \underset{i=1}{\overset{m}{\Pi }}{\left[1-\exp\left(-\zeta \left(x_{\left(i\right)};\beta ,\theta \right)\right)\right]}^{-1}, \end{array}$$

where

$$b_{1}^{*}\left(\beta ,\theta \right)=b_{1}-\left(n-m\right)\log\left[1-\exp\left(-\zeta \left(x_{\left(m\right)};\beta ,\theta \right)\right)\right]-\sum_{i=1}^{m}\log\left[1-\exp\left(-\zeta \left(x_{\left(i\right)};\beta ,\theta \right)\right)\right]$$

and $A=\int_{0}^{\infty }\int_{0}^{\infty }\int_{0}^{\infty }\;\pi \left(\alpha ,\beta ,\theta |x\right)\;d\alpha \;d\beta \;d\theta$ refers to the normalizing constant of (44).

Then, the BEs of any function of α, β, and θ, say φ(α,β,θ), under the SEL function follows by the posterior expectation of φ(α,β,θ), and it has the form

(45) $$\tilde{\varphi }\left(\alpha ,\beta ,\theta \right)=E\left[\left.\varphi \left(\alpha ,\beta ,\theta \right)\right|x\right]=\frac{1}{A}\int_{0}^{\infty }\int_{0}^{\infty }\int_{0}^{\infty }\varphi \left(\alpha ,\beta ,\theta \right)\;\pi \left(\alpha ,\beta ,\theta |x\right)\;d\alpha \;d\beta \;d\theta .$$

Based on (45), the BEs cannot be obtained in closed forms. Hence, the Markov chain Monte Carlo (MCMC) techniques are adopted to approximate the BEs and to construct the CIs from (45). The Metropolis Hastings (M-H) algorithm is a general technique of a family of Markov chain (MC) simulation methods, and it is the most commonly used of MCMC techniques to draw samples from posterior distribution (PD) to calculate the Bayesian estimates of interest. Several applications of the MC algorithm can be explored in [31,32].

Using (45), the full conditional posterior distributions (CPDs) of α, β, and θ are obtained as

(46) $$\begin{array}{c} \pi_{1}\left(\alpha |\beta ,\theta ,x\right)\propto \alpha^{m+a_{1}-1}e^{-\alpha b_{1}^{*}\left(\beta ,\theta \right)}, \end{array}$$

(47) $$\begin{array}{c} \pi_{2}\left(\beta |\alpha ,\theta ,x\right)\propto \beta^{m+a_{2}-1}e^{-b_{2}\beta }\underset{i=1}{\overset{m}{\Pi }}{\left[1-\exp\left(-\zeta \left(x_{\left(i\right)};\beta ,\theta \right)\right)\right]}^{-1} \end{array}$$

and

(48) $$\begin{array}{c} \pi_{3}\left(\theta |\alpha ,\beta ,x\right)\propto \theta^{a_{3}-1}\exp\left(-\theta \left(b_{3}-\sum_{i=1}^{m}x_{\left(i\right)}^{-1}\right)\right)\underset{i=1}{\overset{m}{\Pi }}{\left[1-\exp\left(-\zeta \left(x_{\left(i\right)};\beta ,\theta \right)\right)\right]}^{-1}. \end{array}$$

Thus, from (46), the unknown parameter α has the Ga density with shape parameter $m+a_{1}$ and scale parameter $b_{1}^{*}\left(\beta ,\theta \right)$. Thus, the samples of α are generated easily using any Ga-generating routine. In addition, from (47) and (48), it can be seen that the CPDs of β and θ are different from well-known distributions. Hence, it is impossible to sample directly using standard models. To solve this problem, Tierney [33] proposed the use of a hybrid MCMC algorithm by combining the M-H algorithm sampler with a Gibbs sampling scheme using the normal distribution. Here, the hybrid algorithm will be termed as a M-H within Gibbs sampling for updating the unknown parameter α using Gibbs steps and then for updating the unknown parameters β and θ using M-H steps in order to calculate the BEs and construct the CIs of α, β, and θ. Now, the proposed hybrid algorithm can be carried out using the following steps.

Step 1: Start with an initial values $\alpha^{\left(0\right)}=\hat{\alpha }$, $\beta^{\left(0\right)}=\hat{\beta }$, and $\theta^{\left(0\right)}=\hat{\theta }$.

Step 2: Set J=1.

Step 3: Generate $\alpha^{\left(J\right)}$ from $Ga(m+a_{1},b_{1}^{*}\left(\beta ,\theta \right))$.

Step 4: Generate $\beta^{\left(J\right)}$ and $\theta^{\left(J\right)}$ from $\pi_{2}\left(\left.\beta^{\left(J-1\right)}\right|\alpha^{\left(J\right)},\theta^{\left(J-1\right)},x\right)$ and $\pi_{3}\left(\left.\theta^{\left(J-1\right)}\right|\alpha^{\left(J\right)},\beta^{\left(J\right)},x\right)$ using M-H algorithm with the normal densities:

(a) Generate $\beta^{*}$ and $\theta^{*}$ from $N(\beta^{\left(J-1\right)},\sigma_{\beta }^{2})$ and $N(\theta^{\left(J-1\right)},\sigma_{\theta }^{2})$, respectively.

(b) Obtain the acceptance measures:

$\phi_{1}\left(\beta^{\left(J-1\right)},\beta^{*}\right)=\min\left\{1,\frac{\pi_{2}\left(\left.\beta^{*}\right|\alpha^{\left(J\right)},\theta^{\left(J-1\right)},x\right)}{\pi_{2}\left(\left.\beta^{\left(J-1\right)}\right|\alpha^{\left(J\right)},\theta^{\left(J-1\right)},x\right)}\right\}$ and

$\phi_{2}\left(\theta^{\left(J-1\right)},\theta^{*}\right)=\min\left\{1,\frac{\pi_{3}\left(\left.\theta^{*}\right|\alpha^{\left(J\right)},\beta^{\left(J\right)},x\right)}{\pi_{3}\left(\left.\theta^{\left(J-1\right)}\right|\alpha^{\left(J\right)},\beta^{\left(J\right)},x\right)}\right\}$.

(c) Generate samples $u_{1}$ and $u_{2}$ from U(0,1).

(d) If $u_{1}\leq \phi_{1}$ then set $\beta^{\left(J\right)}=\beta^{*}$. Similarly if $u_{2}\leq \phi_{2}$ then set $\theta^{\left(J\right)}=\theta^{*}$. Otherwise set $\beta^{\left(J\right)}=\beta^{\left(J-1\right)}$ and $\theta^{\left(J\right)}=\theta^{\left(J-1\right)}$.

Step 5: Put J=J+1.

Step 6: Repeat steps 3-5 for *M* times to get

$$\varphi^{\left(J\right)}=\left(\alpha^{\left(J\right)},\beta^{\left(J\right)},\theta^{\left(J\right)}\right),\;J=1,2,\ldots ,M.$$

In the beginning of the analysis (burn-in period), we discarded the first simulated varieties, say $M_{0}$, to remove the effect of the selection of initial guess value and to guarantee the sampling convergence; hence, the remaining samples are used to carried out the BEs with an optimal acceptance rate of 23.4% [34]. Then, for sufficiently large *M*, the drawn MCMC samples of the parameters α, β, and θ as in $\varphi^{\left(J\right)},\;J=M_{0}+1,...,M$, can be adopted to develop the BEs. Thus, the approximate BEs of φ under SEL function takes the form

$$\tilde{\varphi }=\frac{1}{M-M_{0}}\sum_{J=M_{0}+1}^{M}\varphi^{\left(J\right)}.$$

To construct the 100(1−γ)% two-sided Bayes-CIs (BCIs) of α, β, and θ, we order the simulated MCMC samples of $\varphi^{\left(J\right)}$ for J=1,2,…,N, after burn-in as $\varphi_{\left(M_{0}+1\right)},\varphi_{\left(M_{0}+2\right)},\ldots ,\varphi_{\left(N\right)}$. Hence, the 100(1−γ)% two-sided BCIs of φ reduces to

φ(M−M0)γγ22,φ(M−M0)1−γγ22.

## 5. Simulation Results

To evaluate the behavior of the point and interval estimators for the EIGo parameters, we conduct a Monte Carlo simulation study. Using three sets of parametric values, i.e., (α,β,θ)=(0.5,0.1,0.1), (α,β,θ)=(1.0,0.5,0.5), and (α,β,θ)=(3,3,3), we simulate 1000 samples of *n* (total sample size) and *m* (effective sample size) such as n=40, 60, and 80, where *m* is taken as a failure proportion such as (m/n)100%=25, 50, 75, and 100% for each *n*. Clearly, the Type-II censored samples, which are generated with (m/n)=100%, represent the complete samples. In the Bayesian paradigm, the choice of the hyper-parameter value is a crucial issue. Therefore, if the proper prior information (PI) is available for α, β, and θ i.e., $a_{i}=b_{i}=0,\;i=1,2,3$, then the joint posterior distribution (44) is proportional to the likelihood function (35). Hence, if one does not have PI on the unknown parameters, it is better to adopt the MLEs instead of the BEs because the latter are very expensive computationally.

Here, we adopted two informative priors for each set of α, β, and θ, called prior (1): $\left(a_{1},a_{2},a_{3}\right)=\left(1.0,0.2,0.2\right),b_{i}=2,\;i=1,2,3$; prior (2): $\left(a_{1},a_{2},a_{3}\right)=\left(2.5,0.5,0.5\right),$$b_{i}=5,\;i=1,2,3$ when (α,β,θ)=(0.5,0.1,0.1) as well as prior (1): $\left(a_{1},a_{2},a_{3}\right)=\left(2,1,1\right),b_{i}=2,\;i=1,2,3$; prior (2): $\left(a_{1},a_{2},a_{3}\right)=\left(5.0,2.5,2.5\right),b_{i}=5,\;i=1,2,3$ when (α,β,θ)=(1.0,0.5,0.5). Here, the values of hyper parameters of α, β, and θ are determined in such a way that the prior mean becomes the expected value of the estimated parameter [30]. The hybrid MCMC algorithm described in Section 4.2 is adopted to generate 12,000 MCMC samples, and we discarded the first 2000 values as ‘burn-in’. Accordingly, the average Bayes MCMC estimates and 95% two-sided BCIs are calculated based on 10,000 MCMC samples.

For each setting, we compute the average estimates, ${\bar{\hat{\varphi }}}_{k}$, with their root mean squared errors (RMSEs) and RABs using the following formulae.

$$\begin{array}{cc} {\bar{\hat{\varphi }}}_{k} & =\frac{1}{S}\sum_{i=1}^{S}{\hat{\varphi }}_{k}^{\left(i\right)},\;k=1,2,3,\\ \mathrm{RMSE}({\hat{\varphi }}_{k}) & =\sqrt{\frac{1}{S}\sum_{i=1}^{S}{\left({\hat{\varphi }}_{k}^{\left(i\right)}-\varphi_{k}\right)}^{2}},\;k=1,2,3\\ \mathrm{and}\;\mathrm{RAB}({\hat{\varphi }}_{k}) & =\frac{1}{S}\sum_{i=1}^{S}\frac{\left|{\hat{\varphi }}_{k}^{\left(i\right)}-\varphi_{k}\right|}{\varphi_{k}},\;k=1,2,3, \end{array}$$

where *N* is the number of replicates, $\hat{\varphi }$ is an estimate of φ, $\varphi_{1}=\alpha$, $\varphi_{2}=\beta$, and $\varphi_{3}=\theta$.

The required numerical results are performed using the R software. The average values of α, β, and θ, RMSEs, and RABs are reported in Table 1, Table 2 and Table 3. In addition, the average confidence lengths (ACLs) of 95% asymptotic CIs of α, β, and θ are summarized in Table 4.

From Table 1, Table 2 and Table 3, it can be shown that the proposed estimates of the parameters α, β, and θ are very good in terms of minimum RMSEs and RABs. Further, as *n* or *m* increases, the performance of the estimates becomes better. Moreover, the point estimates become even better with the increase in failure-proportion m/n%. Finally, the Bayes MCMC estimates using Ga informative priors are better as they include prior information than the frequentist estimates in term of their RMSEs and RABs. Generally, we conclude that the BEs based on prior (2) performed better than those based on prior (1) in terms of minimum RABs, RMSEs, and ACLs. This is due to the fact that the variance of prior (2) is lower than the variance of prior (1), and both are more informative than an improper prior for $a_{i}=b_{i}=0,\;i=1,2,3$.

Furthermore, the ACLs of asymptotic CIs are narrowed down with the increase in *n* and *m*. In addition, the CIs perform better than the asymptotic intervals due to the Ga prior information with respect to the shortest ACLs. Moreover, when the true values of α, β, and θ increase, it is clear that the associated RMSEs, RABs, and ACLs of all proposed estimates increase. Finally, we recommend the Bayesian MCMC estimation of the parameters of the EIGo distribution using the hybrid Gibbs within the M-H algorithm sampler.

## 6. Real-Life Applications

The importance and flexibility of the EIGo model are discussed empirically by analyzing two real data from engineering science. The first dataset consists of 25 (100 cm) specimens of yarn, which were tested at a certain strain level, and it represents the number of cycles to failure [29,35]. The data are: 20, 15, 61, 38, 98, 42, 86, 76, 146, 121, 157, 149, 175, 180, 176, 180, 220, 198, 224, 264, 251, 282, 325, 321, 653. The second dataset shows the time between failures for repairable mechanical equipment items [36]. The data are: 0.11, 0.30, 0.40, 0.45, 0.59, 0.63, 0.70, 0.71, 0.74, 0.77, 0.94, 1.06, 1.17, 1.23, 1.23, 1.24, 1.43, 1.46, 1.49, 1.74, 1.82, 1.86, 1.97, 2.23, 2.37, 2.46, 2.63, 3.46, 4.36, 4.73.

The EIGo distribution is compared with some competing distributions such as the IGo, IE [21], GIE [20], inverse-Weibull (IW) [37], inverse gamma (IGa) [38], generalized inverse-Weibull (GIW) [39], exponentiated inverted-Weibull (EIW) [40], generalized inverted half-logistic (GIHL) [41], inverted-Kumaraswamy (IK) [42], inverted Nadarajah–Haghighi (INH) [43], and alpha-power inverse-Weibull (APIW) [44] distributions. The corresponding PDFs of the competing models (for x>0) are written in Table 5.

Moreover, to check the validity of the EIGo model along with other competing models, we employed several goodness-of-fit measures as listed in Table 6.

**Figure 3:**
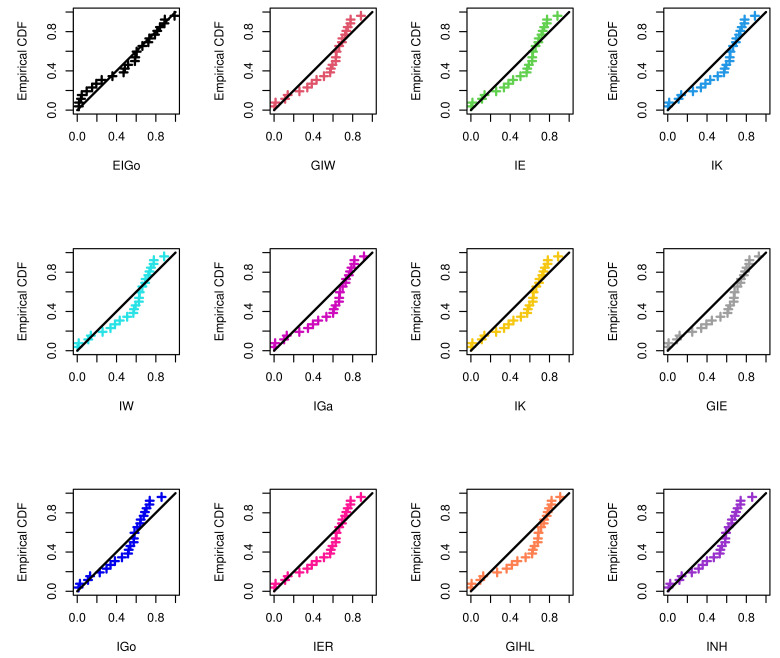
The Q–Q plots of EIGo distribution and its competing models for first data.

**Figure 4:**
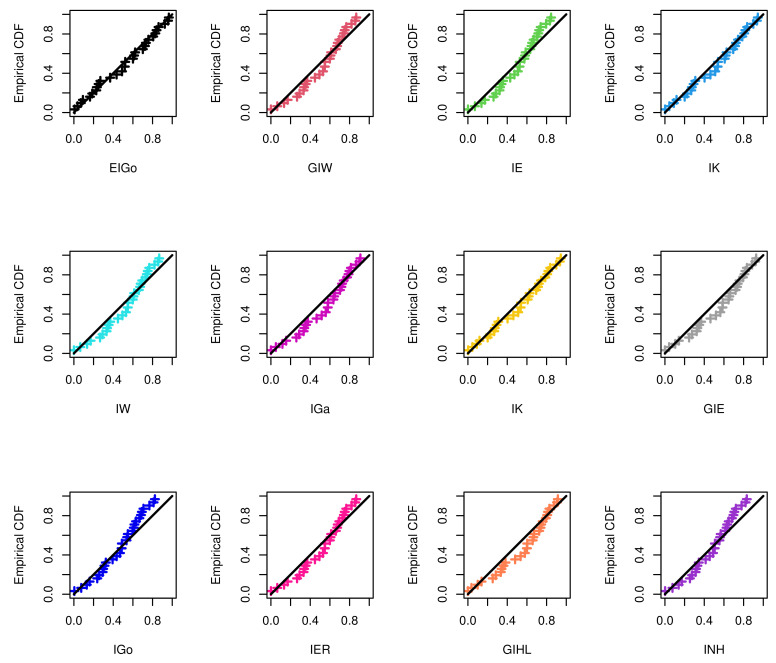
The Q–Q plots of EIGo distribution and its competing models for second data.

The R software and ML approach are adopted to estimate the parameters of the considered distributions and also to evaluate the goodness-of-fit measures. The calculated values of the ML estimates of the model parameters with their standard errors (SEs) and corresponding selection measures, for both data sets, are provided in Table 7 and Table 8, respectively. Moreover, Figure 3 and Figure 4 show graphically the quantile–quantile (Q–Q) plots of all competitive distributions for both datasets.

**Figure 5:**
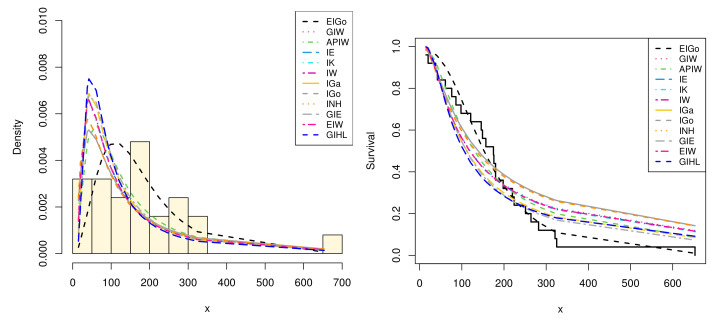
The relative histogram and fitted densities of competing models (**left**) and fitted and empirical SFs (**right**) for first data.

**Figure 6:**
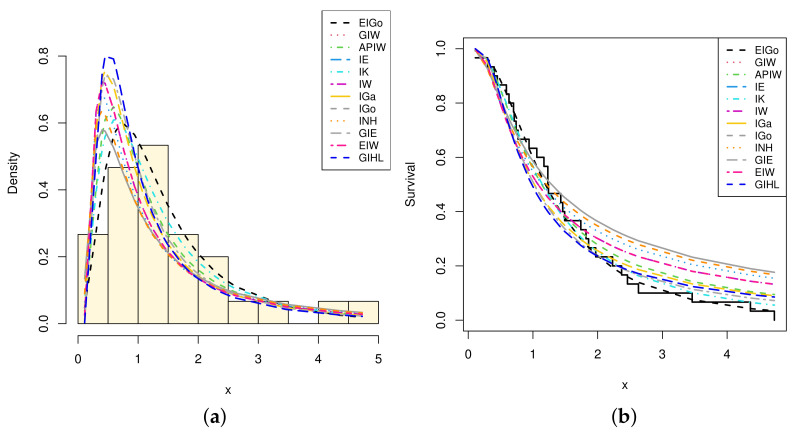
The relative histogram and fitted densities of competing models (**a**) and fitted and empirical SFs (**b**) for second data.

Among all fitted competitive models, Table 7 and Table 8 show that the EIGo distribution has the lowest values of NCL, AIC, CAIC, BIC, HQIC, K-S, A-D, and CvM and the highest *p*-value. Consequently, the EIGo distribution provides better fit, for the given datasets, than the IGo and other inverted distributions. Furthermore, the relative histograms of both datasets and the fitted densities, as well as the plot of fitted and empirical survival functions (SFs), are displayed in Figure 5 and Figure 6, respectively. It is seen that, the graphical presentations in Figure 3, Figure 4, Figure 5 and Figure 6 support the numerical findings.

## 7. Conclusions

In this paper, we have proposed a new three-parameter model called the extended inverse-Gompertz (EIGo) distribution. The EIGo model generalizes some well-known models such as the inverted-exponential, generalized inverted-exponential, and inverse-Gompertz distributions. Various statistical and reliability properties of the EIGo distribution have been addressed. The EIGo parameters have been estimated by the maximum-likelihood and Bayesian approaches under Type-II censoring. The performances of the maximum likelihood and Bayesian estimators have been examined by detailed simulation results. Based on our study, we recommend the Bayesian MCMC estimation of the parameters of the EIGo distribution using the hybrid Gibbs within M-H algorithm sampler. Finally, two real-life engineering data sets have been analyzed to illustrate the applicability of the EIGo distribution as compared with other competing models. The EIGo model provides an adequate and improved fit with respect to its competing inverted models. The failure rate of the EIGo model can only be upside-down-bathtub-shaped. Hence, for future works, the authors suggest that other extensions of the inverse-Gompertz distribution be proposed that may provide all important shapes for the hazard rate including increasing, bathtub, decreasing, and unimodal shapes.

## Figures and Tables

**Table 1:** The average estimates of α and their respective RMSEs and RABs in parentheses.

(α,β,θ)	*n*	*m*	MLE	MCMC	
				Prior (1)	Prior (2)
(0.5,0.1,0.1)	40	40	0.5175 (0.1327,0.1993)	0.5013 (0.0856,0.1338)	0.4884 (0.0728,0.1164)
		30	0.4516 (0.1910,0.3075)	0.4096 (0.1456,0.2435)	0.4150 (0.1356,0.2265)
		20	0.4109 (0.3225,0.4847)	0.2941 (0.2414,0.4326)	0.3505 (0.2135,0.3660)
	60	60	0.5149 (0.1099,0.1693)	0.4888 (0.0618,0.0922)	0.4954 (0.0607,0.0973)
		45	0.4356 (0.1776,0.2799)	0.4034 (0.1391,0.2321)	0.4112 (0.1343,0.2214)
		30	0.4010 (0.2607,0.4204)	0.2959 (0.2366,0.4219)	0.3217 (0.2214,0.3847)
	80	80	0.5086 (0.0892,0.1373)	0.5117 (0.0592,0.0919)	0.5048 (0.0572,0.0901)
		60	0.4369 (0.1671,0.2445)	0.4015 (0.1368,0.2264)	0.4034 (0.1346,0.2221)
		40	0.3924 (0.2393,0.3845)	0.3129 (0.2263,0.3957)	0.3232 (0.2198,0.3791)
(1.0,0.5,0.5)	40	40	1.0778 (0.3812,0.2744)	1.0787 (0.1936,0.1471)	1.0251 (0.1533,0.1191)
		30	1.0040 (0.6560,0.4307)	0.7944 (0.2908,0.2449)	0.8300 (0.2613,0.2162)
		20	1.2427 (1.8647,0.7604)	0.7024 (0.4310,0.3690)	0.6739 (0.4149,0.3573)
	60	60	1.0423 (0.2756,0.2103)	1.0358 (0.1384,0.1074)	1.0081 (0.1224,0.0963)
		45	0.9583 (0.4883,0.3602)	0.8395 (0.2659,0.2175)	0.8252 (0.2587,0.2117)
		30	0.9968 (0.7368,0.5038)	0.6790 (0.4301,0.3682)	0.6804 (0.4167,0.3547)
	80	80	1.0356 (0.2400,0.1773)	1.0170 (0.1138,0.0890)	1.0178 (0.1098,0.0864)
		60	0.9068 (0.3824,0.2994)	0.8297 (0.2577,0.2099)	0.8247 (0.2544,0.2073)
		40	0.9375 (0.5979,0.4590)	0.6725 (0.4279,0.3646)	0.6710 (0.4196,0.3572)

**Table 2:** The average estimates of β and their respective RMSEs and RABs in parentheses.

(α,β,θ)	*n*	*m*	MLE	MCMC	
				Prior (1)	Prior (2)
(0.5,0.1,0.1)	40	40	0.1032 (0.0576,0.4315)	0.0918 (0.0167,0.1389)	0.0894 (0.0127,0.1077)
		30	0.1236 (0.0869,0.6335)	0.0951 (0.0171,0.1404)	0.1050 (0.0138,0.1178)
		20	0.1651 (0.1664,1.1435)	0.0922 (0.0267,0.2333)	0.1143 (0.0230,0.1884)
	60	60	0.1032 (0.0463,0.3600)	0.0908 (0.0094,0.0918)	0.0935 (0.0069,0.0653)
		45	0.1205 (0.0759,0.5598)	0.0997 (0.0116,0.0927)	0.1034 (0.0106,0.0772)
		30	0.1576 (0.1349,0.9860)	0.0899 (0.0185,0.1541)	0.1070 (0.0131,0.1052)
	80	80	0.0999 (0.0377,0.2949)	0.1018 (0.0034,0.0248)	0.0997 (0.0054,0.0465)
		60	0.1173 (0.0645,0.5038)	0.1002 (0.0073,0.0582)	0.1026 (0.0060,0.0510)
		40	0.1573 (0.1213,0.9165)	0.1064 (0.0146,0.1099)	0.1096 (0.0111,0.0964)
(1.0,0.5,0.5)	40	40	0.5255 (0.2675,0.4154)	0.5263 (0.0329,0.0532)	0.4938(0.0204,0.0365)
		30	0.6253 (0.4660,0.6616)	0.4578 (0.0459,0.0843)	0.5156 (0.0234,0.0350)
		20	0.9423 (0.9660,1.2917)	0.5326 (0.0518,0.0764)	0.5254 (0.0333,0.0551)
	60	60	0.5158 (0.2204,0.3440)	0.5124 (0.0136,0.0248)	0.4939 (0.0109,0.0161)
		45	0.6043 (0.3689,0.5509)	0.5225 (0.0323,0.0517)	0.5193 (0.0223,0.0402)
		30	0.8500 (0.7219,1.0446)	0.5405 (0.0454,0.0810)	0.5331 (0.0352,0.0663)
	80	80	0.5118 (0.1879,0.2942)	0.5059 (0.0063,0.0119)	0.5047 (0.0061,0.0109)
		60	0.5871 (0.3089,0.4833)	0.5219 (0.0241,0.0438)	0.5180 (0.0197,0.0360)
		40	0.8212 (0.6443,0.9580)	0.5429 (0.0451,0.0857)	0.5406 (0.0436,0.0812)

**Table 3:** The average estimates of θ and their respective RMSEs and RABs in parentheses.

(α,β,θ)	*n*	*m*	MLE	MCMC	
				Prior (1)	Prior (2)
(0.5,0.1,0.1)	40	40	0.1260 (0.0830,0.6041)	0.1195 (0.0260,0.2072)	0.0920 (0.0098,0.0809)
		30	0.1275 (0.1167,0.7961)	0.1358 (0.0417,0.3606)	0.0940 (0.0111,0.0903)
		20	0.1464 (0.2073,1.3049)	0.0845 (0.0480,0.4256)	0.1240 (0.0284,0.2452)
	60	60	0.1164 (0.0616,0.4733)	0.0942 (0.0064,0.0583)	0.1013 (0.0026,0.0220)
		45	0.1154 (0.0866,0.6312)	0.1070 (0.0142,0.1008)	0.1081 (0.0118,0.0953)
		30	0.1224 (0.1576,1.0183)	0.1009 (0.0217,0.1749)	0.0915 (0.0189,0.1452)
	80	80	0.1142 (0.0512,0.3945)	0.1045 (0.0053,0.0454)	0.1007 (0.0023,0.0192)
		60	0.1124 (0.0826,0.5780)	0.1067 (0.0089,0.0767)	0.0951 (0.0073,0.0567)
		40	0.1047 (0.1233,0.8588)	0.0908 (0.0195,0.1624)	0.1073 (0.0117,0.0864)
(1.0,0.5,0.5)	40	40	0.5831 (0.3268,0.4871)	0.5626 (0.0647,0.1253)	0.5420 (0.0438,0.0840)
		30	0.6044 (0.5502,0.7128)	0.5832 (0.0863,0.1663)	0.4607 (0.0577,0.0917)
		20	0.5877 (0.8856,1.1044)	0.5958 (0.1005,0.1916)	0.4621 (0.0628,0.1061)
	60	60	0.5564 (0.2585,0.3954)	0.5169 (0.0176,0.0338)	0.5057 (0.0117,0.0170)
		45	0.5493 (0.3978,0.5602)	0.5270 (0.0405,0.0649)	0.4786 (0.0273,0.0466)
		30	0.5250 (0.6820,0.8798)	0.5609 (0.0642,0.1217)	0.5461 (0.0494,0.0923)
	80	80	0.5427 (0.2162,0.3345)	0.4927 (0.0082,0.0147)	0.5049 (0.0059,0.0100)
		60	0.5247 (0.3322,0.4831)	0.5110 (0.0141,0.0237)	0.4892 (0.0131,0.0221)
		40	0.4866 (0.5702,0.7752)	0.5479 (0.0517,0.0959)	0.5171 (0.0192,0.0343)

**Table 4:** The ACLs for 95% ACIs/BCIs of α, β, and θ.

			ACI			BCI					
(α,β,θ)	n	m	α	β	θ	α		β		θ	
						**Prior (1)**	**Prior (2)**	**Prior (1)**	**Prior (2)**	**Prior (1)**	**Prior (2)**
(0.5,0.1,0.1)	40	40	0.3485	0.1039	0.0039	0.3352	0.2808	0.0594	0.0215	0.0639	0.0173
		30	0.3807	0.1115	0.0031	0.4424	0.4216	0.0625	0.0467	0.0783	0.0333
		20	0.4208	0.1444	0.0118	0.4760	0.5772	0.0774	0.0696	0.1437	0.0581
	60	60	0.2646	0.0577	0.0019	0.2387	0.2347	0.0092	0.0077	0.0082	0.0088
		45	0.3091	0.1168	0.0033	0.3912	0.3890	0.0463	0.0400	0.0475	0.0321
		30	0.3393	0.1537	0.0053	0.4458	0.4907	0.0632	0.0452	0.0828	0.0695
	80	80	0.2213	0.0294	0.0008	0.2269	0.2228	0.0117	0.0174	0.0103	0.0087
		60	0.2572	0.0918	0.0023	0.3736	0.3674	0.0284	0.0201	0.0229	0.0201
		40	0.2689	0.1219	0.0054	0.4756	0.4753	0.0526	0.0207	0.0676	0.0309
	40	40	0.6913	0.3121	0.0153	0.6838	0.5868	0.0766	0.0673	0.0639	0.0506
		30	0.9376	0.5906	0.0391	0.8035	0.7754	0.0657	0.0670	0.0931	0.1271
		20	1.5049	0.7474	0.0476	1.1633	0.9696	0.1527	0.0868	0.1097	0.1546
	60	60	0.5241	0.1494	0.0051	0.5250	0.4800	0.0202	0.0296	0.0177	0.0361
		45	0.7699	0.5038	0.0213	0.8334	0.7452	0.0887	0.0457	0.0818	0.0631
		30	0.8646	0.5170	0.0182	1.0647	0.9860	0.0740	0.0481	0.0791	0.0753
	80	80	0.4425	0.1082	0.0041	0.4426	0.4213	0.0089	0.0148	0.0114	0.0106
		60	0.5357	0.3158	0.0094	0.7461	0.7131	0.0371	0.0248	0.0292	0.0243
		40	0.7310	0.4141	0.0098	1.0136	0.9521	0.0512	0.0514	0.0608	0.0342

**Table 5:** Some competing inverted models of the EIGo distribution.

Molel	PDF
IE	$f\left(x\right)=\theta x^{-2}\exp\left(-\theta /x\right)$
IW	$f\left(x\right)=\beta \theta x^{-(\beta +1)}\exp\left(-\theta x^{-\beta }\right)$
GIE	$f\left(x\right)=\beta \theta x^{-2}\exp\left(-\theta /x\right){\left[1-\exp(-\theta /x)\right]}^{\beta -1}$)
IGa	$f\left(x\right)=\frac{\theta^{\beta }}{\Gamma (\beta )}x^{-\beta -1}\exp\left(-\theta /x\right)$
GIW	$f\left(x\right)=\alpha \beta \theta^{\beta }x^{-\beta -1}\exp\left(-\alpha {\left(\theta /x\;\right)}^{\beta }\right)$
EIW	$f\left(x\right)=\beta \theta x^{-\beta -1}{\left[\exp\left(-x^{-\beta }\right)\right]}^{\theta }$
GIHL	$f\left(x\right)=2\beta \theta^{-1}x^{-2}e^{-{\left(\theta x\right)}^{-1}}{\left[1-e^{-{\left(\theta x\right)}^{-1}}\right]}^{\beta -1}{\left[1+e^{-{\left(\theta x\right)}^{-1}}\right]}^{-\beta -1}$
IK	$f\left(x\right)=\alpha \beta {\left(1+x\right)}^{-\beta -1}{\left[1-{\left(1+x\right)}^{-\beta }\right]}^{\alpha -1}$
INH	$f\left(x\right)=\beta \theta x^{-2}{\left(1+\theta x^{-1}\right)}^{\beta -1}\exp\left(1-{\left(1+\theta /x\right)}^{\beta }\right)$
APIW	$f\left(x\right)=\alpha \beta \log\left(\theta \right){\left(\theta -1\right)}^{-1}x^{-(\alpha +1)}\exp\left(-\beta \left(x^{-\alpha }\right)\right)\theta^{\exp(-\beta \left(x^{-\alpha }\right))}$

**Table 6:** Some useful criteria for model selection.

Measure or Criterion (C)	Abbreviation
negative log-likelihood	NLC
Akaike information	AIC
consistent Akaike information	CAIC
Bayesian information	BIC
Hannan-Quinn information	HQIC
Kolmogorov-Smirnov	K-S
Anderson-Darling	A-D
Cramér von Mises	CvM
K-S *p*-value	*p*-value

**Table 7:** The estimates, SEs, and selection measures of the EIGo distribution and other competing models for first data.

Model	Estimates (SEs)			Statistics							
	α	β	θ	NCL	AIC	CAIC	BIC	HQIC	K-S (*p*-Value)	A-D	CvM
EIGo	5.8897 (3.3256)	418.743 (151.19)	70.375 (25.031)	155.330	316.659	317.802	320.316	317.674	0.114 (0.901)	0.4141	0.0696
GIW	1.0110 (0.1412)	6.8390 (92.567)	12.370 (169.25)	158.579	323.158	324.301	326.815	324.172	0.211 (0.215)	1.5866	0.2803
APIW	39.651 (76.857)	1.3072 (0.1886)	115.74 (104.40)	156.569	319.137	320.280	322.794	320.151	0.189 (0.336)	1.2512	0.2226
IE	-	-	82.841 (16.568)	158.582	319.841	319.338	320.383	319.205	0.207 (0.234)	1.5774	0.2787
IK	1.0282 (0.1472)	94.040 (57.964)	-	158.484	320.967	321.513	323.405	321.643	0.213 (0.208)	1.5734	0.2780
IW	-	1.0118 (0.1414)	86.718 (50.775)	158.579	321.158	321.703	323.596	321.834	0.211 (0.215)	1.5873	0.2804
IGa	-	1.2166 (0.3081)	100.65 (31.349)	158.294	320.588	321.133	323.025	321.264	0.241 (0.110)	1.5801	0.2791
IGo	-	101.08 (26.705)	14.535 (14.283)	158.001	320.003	320.548	322.440	320.679	0.222 (0.169)	1.2406	0.2203
INH	-	0.7552 (0.2303)	134.21 (76.920)	158.208	320.416	320.962	322.854	321.093	0.218 (0.186)	1.3574	0.2400
GIE	-	1.3462 (0.3991)	100.68 (26.199)	158.090	320.181	320.726	322.618	320.857	0.249 (0.091)	1.5393	0.2720
EIW	-	86.324 (50.423)	1.0108 (0.1411)	158.579	321.158	321.703	323.596	321.834	0.211 (0.216)	1.5864	0.2802
GIHL	-	0.9758 (0.2592)	0.0089 (0.0020)	160.087	324.175	324.720	326.612	324.851	0.263 (0.062)	1.8631	0.3307

**Table 8:** The estimates, SEs, and selection measures of the EIGo distribution and other competing models for second data.

Model	Estimates (SEs)			Statistics							
	α	β	θ	NCL	AIC	CAIC	BIC	HQIC	K-S (*p*-Value)	A-D	CvM
EIGo	3.5359 (1.4251)	2.3986 (0.7152)	0.3749 (0.1605)	40.768	87.536	88.459	91.739	88.880	0.089 (0.971)	0.1762	0.0286
GIW	1.0730 (0.1314)	0.0761 (0.8851)	11.920 (148.78)	46.376	98.751	99.674	102.95	100.10	0.134(0.656)	1.0815	0.1634
APIW	99.979 (157.11)	1.4079 (0.1745)	0.1922 (0.0751)	43.188	92.376	93.300	96.580	93.721	0.113 (0.836)	0.6445	0.0977
IE	-	-	0.7932 (0.1448)	46.533	95.066	95.209	96.467	95.514	0.160 (0.423)	1.0040	0.1509
IK	2.4609 (0.4214)	4.1716 (1.2783)	-	41.238	86.476	86.921	89.279	87.373	0.111 (0.852)	0.3324	0.0495
IW	-	1.0730 (0.1314)	0.7518 (0.1570)	46.376	96.751	97.196	99.554	97.648	0.134 (0.656)	1.0814	0.1634
IGa	-	1.4211 (0.3327)	1.1272 (0.3153)	45.507	95.015	95.459	97.817	95.911	0.158 (0.445)	1.0080	0.1516
IGo	-	0.9290 (0.2107)	0.1091 (0.1068)	45.920	95.839	96.284	98.642	96.737	0.192 (0.216)	0.6357	0.0956
INH	-	0.8517 (0.2346)	1.0344 (0.5127)	46.370	95.740	97.185	99.543	97.637	0.179 (0.295)	0.8525	0.1270
GIE	-	1.6681 (0.4724)	1.0975 (0.2480)	44.966	93.931	94.376	96.734	94.828	0.163 (0.401)	0.9099	0.1361
EIW	-	0.7518 (0.1570)	1.0730 (0.1314)	46.376	96.751	97.196	99.554	97.648	0.134 (0.656)	1.0814	0.1634
GIHL	-	1.2154 (0.3164)	0.7959 (0.1626)	46.828	97.657	98.101	100.46	98.553	0.179 (0.291)	1.1995	0.1855

## Data Availability

Not applicable.
